# Juvenile Hemochromatosis: A Case Report and Review of the Literature

**DOI:** 10.3390/ph13080195

**Published:** 2020-08-15

**Authors:** Akiyoshi Takami, Yasuaki Tatsumi, Katsuhisa Sakai, Yasumichi Toki, Katsuya Ikuta, Yuka Oohigashi, Junko Takagi, Koichi Kato, Kazuhisa Takami

**Affiliations:** 1 Department of Internal Medicine, Division of Hematology, Aichi Medical University School of Medicine, Nagakute 480-1195, Aichi, Japan; oohigashi.yuka.544@mail.aichi-med-u.ac.jp; 2 Department of Internal Medicine, Kizawa Memorial Hospital, Minokamo 505-8503, Gifu, Japan; k-sakai@kizawa-memorial-hospital.jp (K.S.); k-takami@kizawa-memorial-hospital.jp (K.T.); 3 Laboratory of Medicine, Aichi Gakuin University School of Pharmacy, Nagoya 464-8650, Aichi, Japan; ytatsumi@dpc.agu.ac.jp (Y.T.); kkato@dpc.agu.ac.jp (K.K.); 4 Division of Gastroenterology and Hematology/Oncology, Department of Medicine, Asahikawa Medical University, Asahikawa 078-8510, Hokkaido, Japan; toki@asahikawa-med.ac.jp (Y.T.); ikuta@asahikawa-med.ac.jp (K.I.); 5 Department of Internal Medicine, Division of Endocrinology and Metabolism, Aichi Medical University School of Medicine, Nagakute 480-1195, Aichi, Japan; jutakagi@icloud.com

**Keywords:** hemochromatosis, hepcidin, hemojuvelin

## Abstract

Juvenile hemochromatosis (JH), type 2A hemochromatosis, is a rare autosomal recessive disorder of systemic iron overload due to homozygous mutations of *HJV* (*HFE2*), which encodes hemojuvelin, an essential regulator of the hepcidin expression, causing liver fibrosis, diabetes, and heart failure before 30 years of age, often with fatal outcomes. We report two Japanese sisters of 37 and 52 years of age, with JH, who showed the same homozygous *HJV* I281T mutation and hepcidin deficiency and who both responded well to phlebotomy on an outpatient basis. When all reported cases of JH with homozygous *HJV* mutations in the relevant literature were reviewed, we found—for the first time—that JH developed in females and males at a ratio of 3:2, with no age difference in the two groups. Furthermore, we found that the age of onset of JH may depend on the types of *HJV* mutations. In comparison to patients with the most common G320V/G320V mutation, JH developed earlier in patients with L101P/L101P or R385X/R385X mutations and later in patients with I281T/I281T mutations.

## 1. Introduction

Hereditary hemochromatosis is a rare disorder of systemic iron overload due to deficiency of hepcidin, leading to intestinal iron hyperabsorption, and occurs in an autosomal recessive manner except for autosomal dominant type 4 hemochromatosis [1,2,3,4]. Type 2A hemochromatosis is caused by homozygous mutations of *HJV* (*HFE2*), which encodes hemojuvelin, an essential regulator of the hepcidin expression. Unlike other hereditary hemochromatosis—which mostly occurs in individuals of >30 years of age, rarer subtypes of *HJV*-related type 2A and *HAMP* (encoding hepcidin)-related type 2B hemochromatosis, which usually develop in the first to third decades of life—causes severe clinical complications, such as liver fibrosis, hepatocellular carcinoma, diabetes, hypogonadism, and heart failure; *HJV*-related type 2A hemochromatosis is referred to as juvenile hemochromatosis (JH) [1,5]. 

The first line of therapy in hemochromatosis is iron removal by phlebotomy to prevent the progression of organ damage due to iron overload [4]. However, it has been suggested that iron removal by phlebotomy is generally insufficient for the treatment of JH. Although iron chelating agents, including orally administered agents that promote the mobilization and excretion of iron, have also been reported to be effective for removing iron, these agents may be associated with increased rates of adverse effects in comparison to phlebotomy [2,3,4]. Although substitutive hepcidin and agents stimulating the expression of hepcidin, such as antisense oligonucleotides and ferroportin antagonists, are also under development and are expected to be effective [6,7,8], these drugs are not clinically available at the present time. In order to develop safe and effective treatments for JH, it is important to better understand its clinical features.

We report the cases of two Japanese sisters with JH who showed the same homozygous *HJV* I281T mutation. Both patients showed a middle-aged onset and an indolent clinical course. In addition to these cases, homozygous *HJV* I281T has only been identified in one Greek patient of 39 years of age [9], raising the question whether the development and progression of JH may depend on the type of *HJV* mutation. In order to solve this question, we reviewed all reported cases of JH with homozygous mutations in *HJV*.

## 2. Results and Discussion

### 2.1. Case Report

The proband (patient #1) was a 38-year-old woman, who was referred to us due to a 1-year history of amenorrhea, thirst, and weight loss. The patient’s laboratory findings showed high levels of transferrin saturation (93%), serum ferritin (2274 ng/mL) and alanine aminotransferase (51 U/L; reference range, 5–40), insulin-dependent diabetes, iron deposition of the anterior pituitary gland on nuclear magnetic resonance (NMR), with hypogonadism, and massive iron deposition in hepatocytes observed in a liver biopsy (Table 1). Phlebotomy effectively improved iron overload as a treatment of hemochromatosis to lead to a serum ferritin level <50 ng/mL and has been continued for more than 19 years on an outpatient basis. Patient #2 was a 55-year-old woman and the elder sister of patient #1. Regardless of the diagnosis of hemochromatosis in patient #1 (proband), she was referred to us due to liver dysfunction, dyslipidemia, and hyperglycemia for 3 years and thirst for 1 year. She was also found to have high levels of transferrin saturation (92%), serum ferritin (4340 ng/mL) and alanine aminotransferase (51 U/L), insulin-dependent diabetes, iron deposition of the pituitary gland was observed by NMR, with hypogonadism, and massive iron deposition in hepatocytes detected by liver biopsy, and cardiomyopathy with congestive heart failure, revealing the presence of hereditary hemochromatosis. Phlebotomy successfully improved the organ damage associated with iron overload, resulting in a serum ferritin level <50 ng/mL and has been continued for more than 6 years on an outpatient basis. In both cases, the annual medical check-up did not point to abnormal function of organs including the liver.

### 2.2. Sequence Analysis of HJV and Family Tree

**Figure 1:**
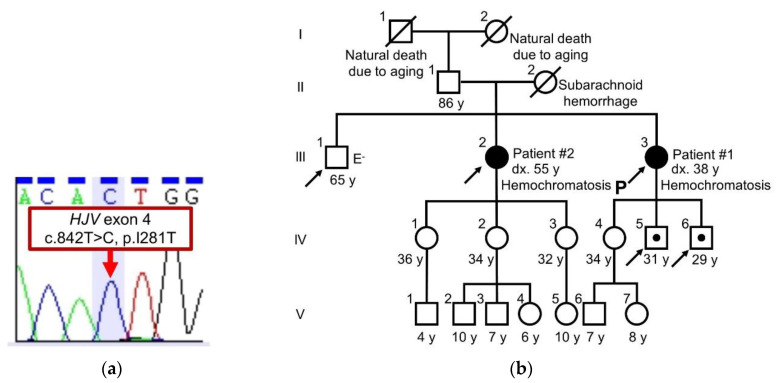
The sequence analysis of *HJV* exon 4 of the proband (patient #1) and the family tree. The homozygous missense mutation c.842T > C, p.I281T (red arrow) is shown. (**a**). The proband (P: patient #1) and the elder sister (patient #2) developed hemochromatosis, while the other family members showed no manifestations of hemochromatosis. (**b**). III-2 and -3, *HJV* I281T/I281T; IV-5 and -6, *HJV* I281T/-. Arrows represent individuals who received a genetic analysis.

Two Japanese siblings were found to have JH with a homozygous mutation of *HJV* exon 4 (c.842 T > C; p.I281T; rs74315326; Figure 1a), and their serum levels of hepcidin were both below the limit of detection (Table 1). With the exception of the two patients, no individuals in the patients’ family presented manifestations of hemochromatosis (Figure 1b). Two sons (IV-5 and IV-6) of the proband who had a monoallelic *HJV* I281T did not show the phenotype of hemochromatosis, which was consistent with previous reports [10,11]. The serum levels of hepcidin in IV-5 and IV-6 were not measured.

### 2.3. All Reported Cases of Juvenile Hemochromatosis

As of July 1, 2020, 96 cases of JH with homozygous *HJV* mutations have been reported in 86 families (Table 2). Cases with compound heterozygous mutations of the *HJV* gene with non-*HJV* genes were excluded from this literature review. The median age at onset and age at the diagnosis in the 96 cases was 25 years (range, 4–60) and 26 years (range, 5–68), respectively. The median time from the onset to the diagnosis of JH was 4 years (range, 0–33). The median age at onset in females and males was 23 years (range, 5–60) and 26 years (range, 5–68) years, respectively, and the median age at the diagnosis was 26 years, regardless of sex (range, 5–68 in females and 16–51 in males), with no significant difference in either the age at the onset or the at the diagnosis. In addition, the female–male ratio of patients with JH was approximately 3:2 (58% *vs.* 42%). These findings may be in contrast to those of *HFE*-associated type 1 hemochromatosis, the most common subtype of hereditary hemochromatosis, which occurs equally in both sexes, in which the onset in females is later than that in males, possibly due to the loss of iron through menstrual bleeding in females [1,12]. The age at the onset of JH did not differ between males and females, while females appeared to be more susceptible to the development of JH. One plausible explanation for these findings is that female hormones may promote iron overload in individuals with the homozygous *HJV* mutation. This hypothesis may be supported by a study [13] that showed that estradiol promoted iron absorption by directly repressing hepcidin transcription in the liver using a mouse model of hemochromatosis. Females with the homozygous *HJV* mutations who were <30 years of age, who are prone to develop JH, may have high estradiol levels [14] and may, therefore, be more susceptible to the suppression of hepcidin than males, leading to the predominance of JH in females. However, in order to prove this hypothesis, the existence of asymptomatic carriers with homozygous *HJV* mutations and the predominance of these asymptomatic carriers in males must be proven, but these have not been identified.

### 2.4. Characteristics of Juvenile Hemochromatosis with Homozygous Identical Mutations in HJV

Table 3 shows the sex and age of cases of JH with homozygous identical mutations in *HJV* in which 3 or more cases have been reported. When the patients—divided into eight groups according to the *HJV* mutations—were compared, using the group with the most frequent mutations of G320V/G320V (median age, 26 years) as a reference, the group with L101P/L101P mutations (median age, 13 years; *p* = 0.004) and the group with R385X/R385X mutations (median age, 15 years; *p* = 0.031) were significantly younger, and the group with I281T/I281T mutations (as observed in the current study) was significantly older (median age, 39 years; *p* = 0.010). Meanwhile, the median age of four cases of JH heterozygous for a single allele of *HJV* I281T, along with *HJV* C321X, C208X, and R6S mutations (Table 2), was 27 years, which was significantly younger in comparison to the group with I281T/I281T (*p* = 0.045) and was not significantly different from the group with G320V/G320V (*p* = 0.76). Accordingly, *HJV* I281T homozygosity may be necessary to delay the onset of JH. There were no significant sex differences among the eight groups. Aside from the current cases, *HJV* I281T homozygosity has only been identified in one Greek patient (Table 1 and Table 2). This Greek patient developed JH at 39 years of age, was diagnosed at 49 years of age, and was the oldest among 12 cases (median age at onset, 27 years; range, 16–39 years) in a first report on *HJV* mutations in JH [9]. It was suggested that, unlike the typical *HJV* G320V mutation in JH, *HJV* I281T homozygosity may be associated with a middle-aged onset of JH; however, this is highly speculative because of the small number of cases.

The mechanisms through which the types of *HJV* mutation may affect the age of onset remains to be determined. Hemojuvelin is mainly expressed in the skeletal muscle, liver, and heart and has been shown to be an essential regulator of hepcidin from hepatocytes based on mouse model experiments [40,41], which demonstrated that *HJV* knockout results in hemochromatosis through hepcidin deficiency. Membrane-bound hemojuvelin is necessary for hepcidin induction, whereas soluble-form hemojuvelin, cleaved by the serine protease matriptase-2, conversely suppresses the induction of hepcidin [42]. Previous studies [42,43,44] have confirmed that *HJV* mutations, as reported in JH, abolished the induction of hepcidin through two mechanisms: inhibition of the membrane export of hemojuvelin in biosynthesis and the decreased function of hemojuvelin itself, while the release of soluble hemojuvelin was not affected. The extent of these two mechanisms varies depending on the type of *HJV* mutation. It remains unknown how types of the *HJV* mutation suppresses the expression of hepcidin through these mechanisms.

The age of onset in six cases (three males and three females) of JH reported from Japan [19,30], including the current study, was relatively high (median age, 44 years; range, 25–52), with a mostly benign course in which phlebotomy was effective in five of the six cases. Thus, the ethnicity and Japanese lifestyle of preferring vegetables and grains as well as green tea containing catechins that suppress the absorption of iron [45], rather than the type of the *HJV* mutation, might defer the onset of JH.

These results suggest that JH is more likely to occur in women in whom female hormones, especially estradiol, can promote iron overload. This may raise the hypothesis that treatment with estradiol-suppressing drugs would be effective for preventing the onset and progression of JH in individuals with the *HJV* homozygous mutations. Furthermore, it was shown that the type of *HJV* mutations may influence the age of onset of JH, suggesting that the sequencing of *HJV* may help predict the progression of organ damage. Besides, analyzing the function of *HJV* mutations may lead to the development of specific targeted therapies for JH.

### 2.5. Limitations

One major limitation of this study is the lack of functional studies supporting the role of homozygous *HJV* mutations, such as *HJV* I281T, in affecting the development of hemochromatosis and, thus, the possibility that other unknown iron overload-related genes may be important causes of hemochromatosis was not excluded.

### 2.6. Strengths of the Present Study

JH is caused by homozygous *HJV* gene mutations and has been thought to occur before 30 years of age, equally in both sexes. However, the current study found—through the second case report of two sibling JH cases with homozygous *HJV* I281T, who showed a middle-aged onset and an indolent course, as well as a review of the relevant literature on JH—that the type of *HJV* mutation and sex may be associated with the development of JH.

## 3. Materials and Methods 

### 3.1. Genetic Analysis for Hemochromatosis

All patients and their family members gave their written informed consent at the time of participating in the molecular studies, in accordance with the Declaration of Helsinki. This project was approved by the Institutional Review Boards of Kizawa Memorial Hospital and Asahikawa Medical University (#15031). After obtaining written informed consent from each individual, blood samples were collected, then genomic DNA purification was performed, and the entire coding regions and splicing junctions of *HFE*, *HFE2 (HJV)*, *HAMP*, *TFR2*, and *SLC40A1* were sequenced according to a previous report [46]. Genetic mutations were then evaluated by comparing the sequencing results to open-access genetic information on the NCBI website. Serum hepcidin was measured by LC-MS/MS, as described previously [47], in findings provided by Medical Care Proteomics Biotechnology Co., Ltd. (Ishikawa, Japan).

### 3.2. Statistical Analyses

The chi-squared test and Mann–Whitney test were used to compare the two groups. For the univariate analysis, two-tailed *p* values of <0.05 were considered to indicate statistical significance. 

## 4. Conclusions

The current case report and review of the relevant literature revealed, for the first time, that JH developed in females and males at a ratio of 3:2, with no age difference between the two groups, and suggested that the age of onset of JH may depend on the type of *HJV* mutations. These findings may lead to the elucidation of the pathophysiology of JH and the development of novel therapeutic strategies.

## Figures and Tables

**Table 1:** Characteristics of patients with homozygous I281T mutations in *HJV* (*HFE2*): the current and reported cases.

No.	1	2	3
Family no.	1	2	2
Ref	[9]	Current case	Current case
Origin	Greece	Japan	Japan
Allele	I281T/I281T	I281T/I281T	I281T/I281T
Age at onset	39	37	52
Age at Dx	49	38	55
Sex	NA	F	F
Years post-Dx	NA	>19	>6
Serum ferritin (ng/mL)	4127	2274	4340
TSAT	90%	93%	92%
Hepcidin (ng/mL)	NA	BDL	BDL
Hypogonadism	+	+	+
Arthropathy	+	+	-
Skin pigmentation	+	+	+
Glucose intolerance	-	+	+
Heart disease	-	-	+
Hepatic fibrosis	+	+	+

Ref, reference; Dx, diagnosis; TSAT, transferrin saturation; F, female; M, male; +, present; -, absent; NA, not available; BDL, below detection limit.

**Table 2:** Characteristics of patients with homozygous *HJV* mutations based on a review of the literature. Please note that cases with compound heterozygous mutations of the *HJV* gene with the non-*HJV* gene were excluded from this review.

No.	Family No.	Ref	Origin	Allele	Age at Onset	Age at Dx	Sex
1	1	[9]	Greece	I281T/I281T	39	49	NA
2	2	Current case	Japan	I281T/I281T	37	38	F
3	2	Current case	Japan	I281T/I281T	52	55	F
4	3	[10]	China	I281T/C321X	14	19	F
5	4	[11]	China	I281T/C321X	26	26	M
6	4	[11]	China	I281T/C321X	27	27	M
7	5	[15]	China	I281T/C208X/R6S	35	36	F
8	6	[9]	Canada	I222N/G320V	7	7	NA
9	7	[9]	Greece	G320V/G320V	21	25	NA
10	8	[9]	Greece	G320V/G320V	32	39	NA
11	9	[9]	Greece	G320V/G320V	25	32	NA
12	10	[9]	Greece	G320V/G320V	20	21	NA
13	11	[9]	Greece	C361fsX366/C361fsX366	26	33	NA
14	12	[9]	Greece	G99V/G99V	28	33	NA
15	13	[9]	Greece	G320V/G320V	21	25	NA
16	14	[9]	Greece	G320V/R326X	33	37	NA
17	15	[9]	Greece	G320V/G320V	29	31	NA
18	16	[9]	France	G320V/G320V	16	23	NA
19	17	[11]	China	C321X/H104R	18	NA	M
20	18	[11]	China	C321X/V274M	57	NA	M
21	19	[11]	China	Q312X/Q312X	22	NA	F
22	20	[11]	China	F103L/F103L	36	NA	F
23	21	[16]	France (Caucasian)	G320V/G320V	28	NA	M
24	22	[16]	France (Caucasian)	G320V/G320V	31	NA	M
25	23	[16]	France (North African)	R385X/R385X	8	NA	M
26	24	[16]	France (Caucasian)	H180R/L101P	60	NA	F
27	25	[16]	France (Caucasian)	A384V/R288W	32	NA	F
28	26	[16]	France (Caucasian)	G320V/G320V	16	NA	F
29	27	[17]	African American	R54X/R54X	4	23	M
30	28	[18]	Romania	G66X/G66X	25	25	M
31	29	[19]	Japan	Y150C/V274M	25	39	M
32	30	[20]	United States	C80R/L101P	18	23	F
33	31	[20]	United States	C80R/L101P	17	21	F
34	31	[20]	United States	L101P/L101P	13	23	F
35	31	[20]	United States	L101P/L101P	15	21	M
36	31	[20]	United States	L101P/L101P	12	18	F
37	31	[20]	United States	L101P/L101P	8	8	F
38	32	[20]	United States	I222N/G320V	17	23	F
39	33	[21]	Bangladesh	C80Y/G320V	NA	19	F
40	34	[21]	Pakistan	G99R/G99R	NA	26	M
41	35	[21]	Pakistan	G99R/G99R	NA	11	F
42	36	[21]	Pakistan	P192L/P192L	NA	23	M
43	37	[21]	Pakistan	L194P/L194P	NA	32	M
44	38	[21]	Sri Lanka	A343PfsX23/A343PfsX23	NA	17	M
45	39	[22]	Australia	G320V/G320V	NA	12	F
46	40	[22]	Australia	C80R/R326X	NA	18	F
47	41	[22]	Australia	G320V/G320V	NA	32	F
48	42	[23]	Italy	R385X/R385X	15	NA	F
49	43	[23]	Italy	F170S/F170S	20	NA	F
50	44	[23]	Italy	W191C/W191C	21	NA	F
51	45	[23]	Italy	R385X/R385X	20	NA	M
52	46	[23]	Italy	D149fsX245/D149fsX245	20	NA	F
53	47	[23]	Italy	S205R/G250V	21	NA	F
54	48	[23]	Italy	F170S/F170S	14	NA	F
55	49	[23]	Italy	V74fsX113/N269fsX311	24	NA	F
56	49	[23]	Italy	D149fsX245/D149fsX245	21	NA	M
57	50	[23]	Italy	R131fsX245/R131fsX245	20	NA	F
58	51	[23]	Canada/Italy	G320V/G320V	29	NA	M
59	52	[23]	Italy	S85P/S85P	30	NA	F
60	53	[23]	France	R288W/R288W	26	NA	F
61	54	[23]	Italy	D172E/G319fsX341	20	NA	F
62	55	[23]	Australia/English	A168D/A168D	28	NA	M
63	56	[23]	Albania	L101P/G99R	26	NA	F
64	57	[23]	Italy	D149fsX245/D149fsX245	22	NA	M
65	58	[23]	Canada/Italy	G320V/G320V	27	NA	F
66	59	[24]	Iran	C89R/C89R	26	26	M
67	59	[24]	Iran	C89R/C89R	30	30	F
68	60	[25]	English/Ireland	G320V/Q116X	25	NA	F
69	61	[26]	Croatia	G320V/G320V	NA	24	M
70	62	[26]	Germany	G320V/G320V	NA	24	M
71	63	[26]	Germany	G320V/G320V	NA	24	M
72	64	[26]	Slovakia	G320V/S328fsX337	NA	25	M
73	64	[26]	Slovakia	G320V/S328fsX337	NA	16	F
74	65	[26]	Germany	G320V/G320V	NA	28	F
75	66	[26]	Germany	C119F/C119F	NA	25	M
76	67	[27]	Netherland	L165X/L165X	NA	16	M
77	68	[28]	France (Caucasian)	R176C/R176C	NA	17	F
78	69	[29]	France	G320V/R176C	5	5	F
79	70	[30]	Japan	D249H/D249H	48	48	M
80	71	[30]	Japan	Q312X/Q312X	51	51	M
81	72	[30]	Japan	Q312X/Q312X	51	51	F
82	73	[31]	Caucasian	G320V/C321W	23	30	F
83	74	[32]	India	D355Y/D355Y	35	42	M
84	74	[32]	India	D355Y/D355Y	28	32	M
85	75	[33]	Romania	G320V/G320V	27	31	F
86	76	[34]	Caucasian	G320V/G320V	20	39	F
87	77	[35]	Italy	C317S/C317S	35	68	F
88	78	[36]	India	G336X/G336X	NA	45	M
89	79	[36]	India	G336X/G336X	NA	49	F
90	80	[36]	India	G336X/G336X	NA	38	F
91	81	[36]	India	G336X/G336X	NA	47	M
92	82	[36]	India	5’UTR -358 (G>A)/ 5’UTR -36 (G>A)	NA	43	M
93	83	[36]	India	5’UTR -358 (G>A)/ 5’UTR -36 (G>A)	NA	32	F
94	84	[37]	China	C321X/Q6H	23	31	M
95	85	[38]	English/Ireland	L28SfsX24/ L28SfsX24	25	25	F
96	86	[39]	Brazil	Q233fsX245/Q233fsX245	26	26	F
							
Total, F/M					49 (58%)/35 (42%)		
Median age (total)					25	26	
Median age (F)					23	26	
Range (F)					5–60	5–68	
Median age (M)					26	26	
Range (M)					4–57	16–51	
*P* value at age (F *vs.* M)					0.47	0.48	
Standard deviation (total)					11	12	
Standard deviation (F)					11	14	
Standard deviation (M)					12	10	

Ref, reference; F, Female; M, Male; NA, not available; 5’UTR, 5’prime untranslated region.

**Table 3:** Relationship of the *HJV* mutation with sex and the age of onset in JH patients with homozygous identical mutations in *HJV* (limited to mutations with ≥3 reported cases).

Group Number	Allele	Origin (Number of Cases)	Number of Cases	Sex, F/M	Median Age, y	Minimum, y	Maximum, y	*p* Value at Age vs. G320V /G320V
**1**	G320V/G320V	Greece (6), France (4), Germany (3), Canada (2), Australia (2), Romania (1), Croatia (1), Caucasian (1)	20	7/6	26	16	32	Reference
**2**	G336X/G336X	India (4)	4	2/2	NA	NA	NA	NA
**3**	L101P/L101P	United states (4)	4	3/1	13	8	15	0.004
**4**	I281T/I281T	Japan (2), Greece (1)	3	2/0	39	37	52	0.010
**5**	Q312X/Q312X	Japan (2), China (1)	3	2/1	51	22	51	0.105
**6**	D149fsX245/D149fsX245	Italy (3)	3	1/2	21	20	22	0.498
**7**	G99R/G99R	Pakistan (2), Greece (1)	3	1/1	28	28	28	0.708
**8**	R385X/R385X	Italy (2), France (1)	3	1/2	15	8	20	0.031

F, female; M, male; NA, not available. Bold typeface represents a significant difference.

## List of Abbreviations

JH	juvenile hemochromatosis
HJV	hemojuvelin
NMR	nuclear magnetic resonance;
Ref	reference
Dx	diagnosis
TSAT	transferrin saturation
F	female
M	male
+	present
-	absent
NA	not available
BDL	below detection limit
P	proband
5’UTR	5’prime untranslated region
